# The Distributional Characteristics of Multiple Sclerosis Lesions on Quantitative Susceptibility Mapping and Their Correlation With Clinical Severity

**DOI:** 10.3389/fneur.2021.647519

**Published:** 2021-07-09

**Authors:** Zhuoxin Guo, Liu Long, Wei Qiu, Tingting Lu, Lina Zhang, Yaqing Shu, Ke Zhang, Ling Fang, Shaoqiong Chen

**Affiliations:** ^1^Department of Radiology, The Third Affiliated Hospital of Sun Yat-sen University, Guangzhou, China; ^2^Department of Neurology, The Third Affiliated Hospital of Sun Yat-sen University, Guangzhou, China

**Keywords:** relapsing–remitting multiple sclerosis, quantitative susceptibility mapping, iron, spatial distribution, clinical outcome

## Abstract

**Background:** Multiple sclerosis (MS) patients have a wide spectrum of severity and responses to therapy; the personalization of treatment relies on sensitive and specific biomarkers. Previous studies have suggested that susceptibility contrast in demyelinated plaques is associated with iron-related pathology in multiple sclerosis which may indicate clinical severity. The aims of this study were to characterize the spatial distribution of MS lesions with different iron patterns by using quantitative susceptibility mapping and to explore neuroradiological findings that correlate with poor clinical outcome.

**Methods:** Twenty-six patients with relapsing–remitting MS [14 men, 12 women; mean age, 29 ± 8 (standard deviation) years; age range, 21–52 years] were included in this study. Differences in lesion number, T2 volume, and susceptibility were compared among lesions subcategorized by location and by the presence or absence of a hyperintense rim on quantitative susceptibility mapping. Associations between these imaging features and clinical outcomes including Expanded Disability Status Scale scores and annual relapse rates were investigated.

**Results:** A total of 811 unifocal MS lesions were included, and their QSM patterns were nodular hyperintensity with no rim (rim–, 540, 67%) or with a hyperintense rim on the edge (rim+, 172, 21%) and nodular isointensity (99, 12%). Rim+ lesions had significantly larger volume (115 ± 142 vs. 166 ± 185 mm^3^, *p* < 0.001) and lower susceptibility (4 ± 15 vs. 8 ± 16 ppb, *p* < 0.05) than rim– lesions. More rim+ lesions were found in periventricular areas [median, 45%; interquartile range (IQR), 36%], whereas a larger proportion of rim– lesions were distributed in juxtacortical (median, 32%; IQR, 21%) and deep white matter (median, 38%; IQR, 22%) areas. The annual relapse rate was positively correlated with the proportion of periventricular rim+ lesions (*p* < 0.001, *r* = 0.65) and the proportion of subtentorial rim+ lesions (*p* < 0.05, *r* = 0.40). Additionally, a significant association was found between the burden of periventricular rim+ lesions (β = 0.64, *p* < 0.001) and the burden of subtentorial rim– lesions (β = 0.36, *p* < 0.05).

**Conclusions:** A high number or lesion burden of periventricular rim+ lesions or subtentorial lesions is associated with frequent clinical relapses.

## Introduction

Multiple sclerosis (MS) is an autoimmune inflammatory disease of the central nervous system that is characterized by diffuse demyelination with neurodegeneration, vasculitis of brain venules, and meningeal inflammation (1–3). The wide spectrum of clinical severity and the variability of prognosis in MS patients highlight the need for personalized medicine, but this undertaking is limited by the lack of sensitive and specific biomarkers. Gd enhancement in MS lesions is an imaging biomarker of blood–brain barrier (BBB) breakdown and inflammatory activity, but its time window is short. Once the BBB is repaired, contrast enhancement is absent even in plaques with continuous demyelination (4). Moreover, correlations between traditional MRI measures and severity in MS patients have been found to be weak in many studies; this problem is known as the “clinicoradiological paradox” (5). Recent research advances on the topic of brain iron hold promise for addressing this issue.

Iron is crucial for myelin formation, synaptic plasticity, oxidative metabolism, and neurotransmitter synthesis (6, 7). Disruption of iron homeostasis has been found in many neurodegenerative disorders (8, 9). In MS, iron accumulation in demyelination lesions is a hallmark of brain tissue injury, while volume loss of deep gray matter could be a consequence of iron depletion from damaged oligodendrocytes, leading to neurodegeneration (10–12). The strong effect of iron on MR phase changes enables *in vivo* iron assessment *via* iron-sensitive imaging [e.g., susceptibility-weighted imaging and quantitative susceptibility mapping (QSM)] (7).

Longitudinal MS studies with QSM demonstrate susceptibility variations in chronic MS lesions, which are probably caused by iron accumulation and clearance (3, 4, 13). In studies correlating histopathology with imaging, iron-laden macrophages with a proinflammatory activation status (rim+ lesions) were found at the rims of slowly expanding demyelinated plaques, showing that an iron rim is indicative of chronic inflammatory activity (14, 15). Moreover, subjects with progressive MS were more likely than those with relapsing–remitting MS (RRMS) to have lesions with iron rims (16, 17). Taken together, these findings reveal that the content and pattern of iron in lesions are associated with MS pathology. Iron concentration has potential as a candidate biomarker to predict prognosis in MS patients. Previous studies have also found links between the density and extent of demyelination and the distance from the surface of the brain and have suggested that lesion localization may play an important role in neurological disability among MS patients (18–20).

Therefore, the aims of this study were to characterize the spatial distribution of MS lesions with different patterns of iron deposition and to explore neuroradiological findings that correlate with poor clinical outcome.

## Materials and Methods

### Patients

We prospectively enrolled 28 patients with clinically confirmed RRMS from the Department of Neurology at our institution. All patients underwent MRI examination. Two patients were excluded due to motion artifacts on MRI images, resulting in a sample size of 26 included patients [14 men, 12 women; mean age, 29 ± 8 (standard deviation) years; age range, 21–52 years]. The severity of the clinical disability of the patients was quantified by using the Expanded Disability Status Scale (EDSS) within 48 h of the MR scan. The times of the clinical relapse since the patients began disease-modifying therapy in our institution were retrospectively reviewed to estimate the annual relapse rate (ARR). All procedures performed in this study involving human participants were in accordance with the ethical standards of the institutional research committee and with the 1964 Helsinki Declaration. All patients gave written informed consent.

### Imaging Protocol and Reconstruction

All images in the study were acquired on a 3.0-T MRI scanner (Discovery MR 750; GE Healthcare, Milwaukee, WI, USA) equipped with an eight-channel head coil. Gradient-echo images (GRE) for QSM reconstruction were acquired with a three-dimensional T2^*^-weighted multi-echo spoiled GRE sequence with the following parameters: first echo time (TE) in ms/TE spacing in ms/number of echoes = 3.4/3.5/14, repetition time (TR) = 52.5 ms, flip angle = 20°, field of view = 180 × 180 mm^2^, matrix = 256 × 256, in-plane resolution = 0.7 × 0.7 mm^2^, thickness = 1 mm, and gap between slices = 0. T2-weighted images (T2WI) were acquired with the following parameters: voxel size = 0.7 × 0.7 × 1 mm^3^, field of view = 180 × 180 mm^2^, matrix = 256 × 256, flip angle = 111°, thickness = 1 mm, and no gap between slices. Conventional MR sequences included T2 fluid-attenuated inversion recovery (T2-FLAIR) [TR/TE/inversion time (TI) = 8,400/145/2,100 ms] and T1-FLAIR (TR/TE/TI = 3,500/24/943 ms), each with a slice thickness of 5 mm and no gap between slices.

QSM images were reconstructed using the STI Suite package (21). iHARPERELLA was applied to perform phase unwrapping and background phase removal, with default parameters [iteration number = 40; pad size = (12 12 12); radius = 12 mm]. The iLSQR technique was used to generate susceptibility maps.

### Imaging Analysis

Imaging data were reviewed to (1) record the distributional areas, number, and QSM patterns of the unifocal MS lesions (ovoid-like shapes with discrete borders rather than confluent borders) and (2) quantify the susceptibility values of hyperintense lesions on QSM and their lesion volume on T2WI. Confluent MS lesions were included only in the measurement of individual T2 burden (sum of lesion volume).

An MS lesion was defined as a T2-hyperintense lesion with an axis length of at least 3 mm. Distributional areas included juxtacortical (a maximum distance of 3 mm from the corticomedullary junction), periventricular (abutting the third and lateral ventricles without white matter in between, including the corpus callosum), deep (within white matter but neither periventricular nor juxtacortical), and subtentorial white matter areas (16, 22). The QSM signal of the lesions described the signal intensity relative to local white matter. MS lesions with QSM signals higher than those of the normal-appearing white matter (NAWM) were identified as QSM hyperintense lesions (23). Lesions containing a visual QSM hyperintense ring on the edge were defined as rim+ lesions. The lesions were identified and characterized by two neuroradiologists (L.L. and Z.G. with 9 and 5 years of experience, respectively) and verified by consensus in case of disagreement.

The susceptibility value and T2 volume of lesions were estimated by using a region of interest (ROI) semiautomatic tool in Analyze 12.0 (AnalyzeDirect, Overland Park, KS, USA). A neuroradiologist (Z.G.) outlined the lesions on the involved slices and revised the outlines manually to exclude veins. Lesion ROIs were drawn on T2WI and QSM separately. The susceptibility of a lesion was defined as the mean of all voxels in the ROI and calculated relative to the susceptibility of central cerebral spinal fluid (CSF). Lateral ventricles were semiautomatically segmented on the first echo of GRE images, and the choroid plexus regions were manually excluded. The ROIs were copied to the QSM to obtain CSF susceptibility. The susceptibility of NAWM relative to CSF was also measured.

### Statistical Analysis

Statistical analysis was conducted using SPSS 21.0. The criterion for statistical significance was set at *p* < 0.05. The following analysis focused on QSM hyperintense lesions.

#### Comparison Between Rim+ and Rim– Lesions

Rim+ and rim– lesions were compared by the Mann–Whitney *U*-test at the lesion level in terms of lesion volume and susceptibility. To investigate the distributional tendency for rim+ and rim– lesions, we used the Friedman test to determine whether the numbers of lesions in four distributional areas were significantly different from each other within subjects; a stepwise step-down test was used for multiple comparisons.

#### Comparison of Lesions in Different Locations

To compare the differences in T2 volume and susceptibility in juxtacortical, deep, and periventricular and subtentorial white matter areas, the Kruskal–Wallis test was used with the Bonferroni correction for multiple comparisons.

#### Relationships Between Imaging Findings and Clinical Outcomes

A mixed-effect regression model was implemented with susceptibility at the lesion level as a response variable and age, sex, individual T2 burden, ARR, and EDSS as covariates. The model also accounted for random factors including intrasubject correlations and intersubject variability in the number of lesions. The model was implemented to assess the associations between the covariates and lesion susceptibility while controlling for rim+ or rim– status as well as lesion locations.

Correlation analysis at the individual level was also performed. Multivariable linear regression was used to evaluate the correlations of individual lesion burden and average lesion susceptibility with clinical outcomes (EDSS and ARR). Candidate variables associated with clinical outcomes at a level of *p* < 0.10 in the univariable analysis were included in the multivariable models. Age and gender were entered as covariates in the models.

Additionally, we divided patients into subgroups by EDSS score (< or ≥4) and ARR (< or ≥1) and evaluated the differences between groups in terms of lesion burden and average lesion susceptibility using the Mann–Whitney *U*-test. The correlation of lesion portion in various locations with clinical outcomes was evaluated by using Spearman's correlation test.

## Results

### Patient and Lesion Characteristics

Clinical characteristics of the MS patients are summarized in Table 1.

**Table 1:** Clinical characteristics of the patients.

	**MS patients (*n* = 26)**
Female, *n* (%)	12 (46%)
Age of onset (years)	Median (range), 22 (9–45)
Disease duration (years)	Median (range), 4 (1–20)
Duration of modifying therapy (years)	Mean ± SD, 2.4 ± 1.2
EDSS score	Median (range, IQR), 2.0 (0–6.5, 3.4)
ARR	Median (range, IQR), 0.7 (0–3.3, 1.2)

*SD, standard deviation; IQR, interquartile range; EDSS, Expanded Disability Status Scale; ARR, annual relapse rate*.

**Figure 1:**
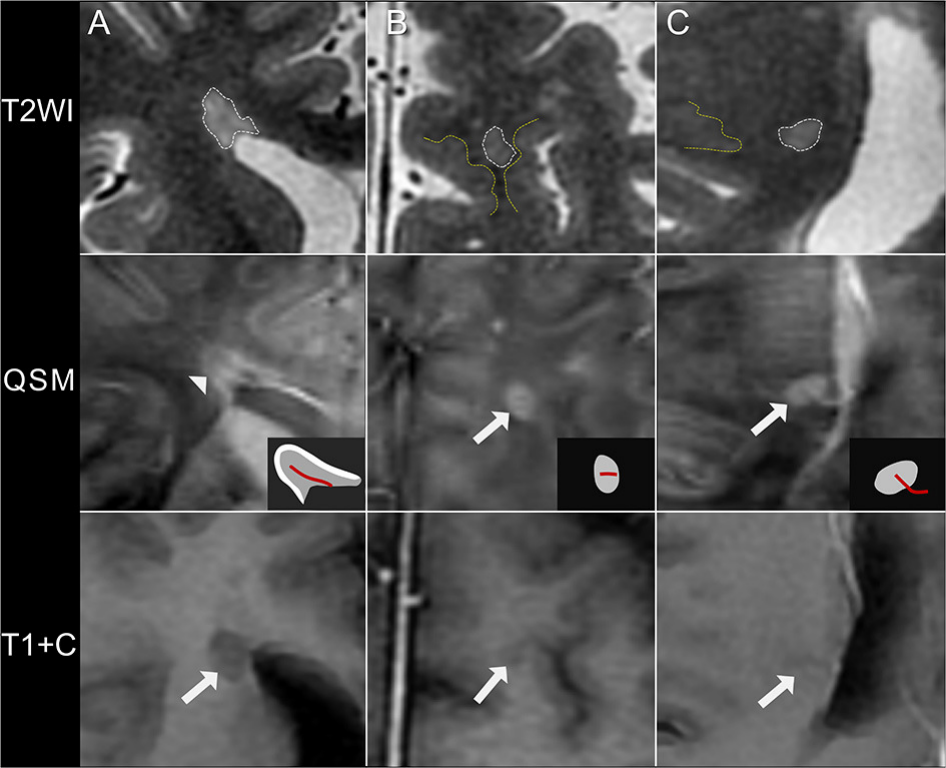
Quantitative susceptibility mapping (QSM) patterns of MS lesions. **(A)** A rim+ periventricular MS lesion. T2WI shows a hyperintense lesion abutting the lateral ventricles without white matter in between. A hyperintense rim is found on the lesion edge on QSM (arrowhead), as illustrated in the diagrams (white region). **(B)** A rim– juxtacortical lesion. T2WI shows a hyperintense lesion located near the cortex, with a distance <3 mm from corticomedullary junction (yellow line). The lesion is nodular hyperintense on QSM with no hyperintense rim (arrow). **(C)** A rim– deep white matter lesion. The lesion is located far away from both the cortex (yellow line) and the lateral ventricle and is hyperintense on QSM with no rim (arrow). These lesions are non-enhancing on axial T1WI after injection of contrast agent (T1+C), suggesting of a chronic status. QSM shows a hyperintense vein passing through the lesion center (the central vein sign), as illustrated in the diagrams (red line).

A total of 840 white matter lesions were detected, with 29 lesions excluded because of poor QSM quality. The QSM contrast patterns of the remaining 811 lesions were nodular hyperintensity with no rim (rim–, 540, 67%) or with a hyperintense rim on the edge (rim+, 172, 21%) and nodular isointensity (99, 12%) (Figure 1). In particular, ring-like zones of hyperintensity were found in the plaque centers of two rim+ lesions, creating the appearance of concentric rings (Supplementary Material).

In our cohort of MS patients, 23 (88%) patients had more rim– hyperintense lesions (*p* < 0.001) than rim+ or isointense lesions. The mean susceptibility of QSM hyperintense lesions was 7.9 ± 13.4 ppb, which was on average 35.4 ± 11.3 ppb higher than that of NAWM (−27.5 ± 11.1 ppb). The mean individual lesion burden was 12,775 ± 11,043 mm^3^.

### Comparison Between Rim+ and Rim– Lesions

Compared with rim– lesions, rim+ lesions had significantly reduced susceptibility values (4 ± 15 vs. 8 ± 16 ppb, *p* < 0.05) and increased T2 volumes (115 ± 142 vs. 166 ± 185 mm^3^, *p* < 0.001). The numbers of rim– lesions in juxtacortical (197, 36%) and deep white matter (192, 36%) areas were higher than the numbers in periventricular (112, 21%) and subtentorial (39, 7%) areas.

### Comparison of Lesions in Different Locations

**Figure 2:**
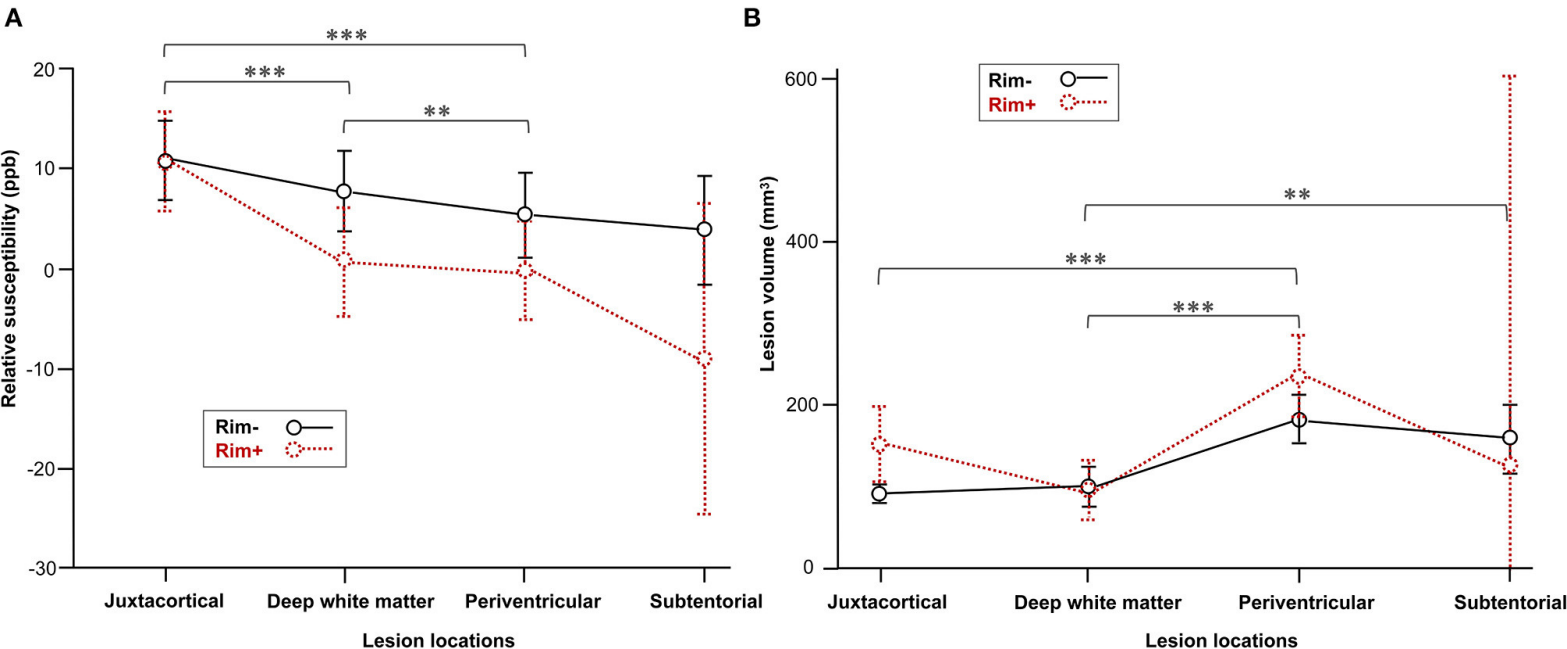
Comparison of MS lesions in different locations. **(A)** Lesion susceptibility in the mixed-effects model after controlling for rim+ vs. rim– status as well as lesion locations. **(B)** Comparison of volume in the rim+ and rim– subgroups among various lesion locations. Data represent the mean (dots) and standard deviation (bars). Adjusted **p < 0.01 and ***p < 0.001indicate significant differences between lesions in different locations.

At the lesion level, susceptibility was highest within juxtacortical lesions (11 ± 15 ppb) (adjusted *p* < 0.01), and lesion susceptibility tended to decrease as the distance from the ventricle decreased (6 ± 16 ppb in deep white matter and 4 ± 16 ppb in the periventricular area). This gradient was not found in NAWM. The average susceptibility was −32 ± 4, −23 ± 2, and −27 ± 1 ppb in juxtacortical, deep, and periventricular NAWM, respectively. The mean volume of periventricular lesions was significantly larger than that of the other supratentorial lesions (adjusted *p* < 0.001) (Figure 2). No significant difference was found between the volumes of periventricular and subtentorial lesions, although the latter had a lower mean.

More rim+ lesions were found in periventricular (63, 37%) and juxtacortical (64, 37%) areas than in deep white matter (42, 24%), whereas only three lesions (2%) were found in subtentorial areas. Sixteen patients had subtentorial rim lesions, while only three patients had rim+ subtentorial lesions. At the individual level, our study also demonstrated significant differences in rim– lesion numbers in four distributional areas and rim+ lesion numbers (both *p* < 0.001). More rim lesions were located in the deep white matter area (median, 38%; IQR, 22%) than in the periventricular (median, 18%; IQR, 21%) or subtentorial (median, 5%; IQR, 13%) areas (*p* < 0.05). Additionally, larger proportions of rim lesions were found in the juxtacortical white matter (median, 32%; IQR, 21%) than in the periventricular and subtentorial areas, but the results of multiple comparison testing were not significant. Furthermore, more rim+ lesions were detected in the periventricular area (median, 45%; IQR, 36%) than in the juxtacortical area (median, 26%; IQR, 44%) or deep white matter (median, 24%; IQR, 29%), although the differences between groups were not significant.

### Relationships Between Imaging Findings and Clinical Outcomes

In the mixed-effects model, lesion susceptibility had a positive association with individual lesion burden (*p* = 0.02), while there was no significant association found with age, sex, EDSS score, or ARR. The mixed-effects model also confirmed significant differences in susceptibility values among rim+ and rim– lesions in various locations (Figure 2).

We found positive correlations of the annual relapse rate with the proportion of periventricular rim+ lesions (*p* < 0.001, *r* = 0.65) and the proportion of subtentorial rim+ lesions (*p* < 0.05, *r* = 0.40) (Table 2). No significant correlation was found between the proportion of different types of lesions in patients and EDSS. Patients with subtentorial rim+ lesions had a higher EDSS score (3.93 ± 2.09) and ARR (1.85 ± 0.21) than those without such lesions. Patients with ARR ≥1 had a significantly higher burden of periventricular rim+ (*p* < 0.05) and rim– (*p* < 0.005) lesions than patients with low ARR. No significant difference was found in the comparison of lesion burden or susceptibility between patients with EDSS scores ≥4 and <4.

**Table 2:** Significant associations between imaging findings and annual relapse rate.

**Individual imaging measures**	***p***	***r* or β**
Proportion of periventricular rim+ lesions	<0.001	*r* = 0.65
Proportion of subtentorial rim+ lesions	<0.05	*r* = 0.40
Burden of periventricular rim+ lesions	<0.001	β = 0.64
Burden of subtentorial rim– lesions	<0.05	β = 0.36

*In Spearman's correlation analysis, the r value is given, and in linear regression analysis, the β value is given*.

Next, we explored the possibility of using the T2 burden and susceptibility of lesions classified by QSM pattern and location as candidate biomarkers predicting clinical outcomes (EDSS and ARR). When ARR was entered as a response, univariable analysis yielded the following candidate variables: the lesion burden of rim– lesions in the juxtacortical, periventricular, and subcortical areas and the lesion burden of rim+ lesions in the periventricular area. In multivariate linear regression analysis with stepwise selection, the burden of periventricular rim+ lesions (β = 0.64, *p* < 0.001) and subtentorial rim– lesions (β = 0.36, *p* < 0.05) remained in the final model (*R*^2^ = 0.59) (Table 2). The regression analysis to predict EDSS did not identify any significant predictors.

## Discussion

The current study reveals high heterogeneity in distribution and susceptibility contrast among MS lesions, which might contribute to the breadth of the clinical spectrum in RRMS patients. A high number or lesion burden of periventricular rim+ lesions or subtentorial lesions is associated with frequent clinical relapses.

In this study, lesions with a hyperintense rim accounted for only 21% of lesions visible on QSM, similar to the figure reported in a previous study (24). Although the number of lesions was small, the lesions were larger in volume than rim– lesions, which is consistent with previous observations (25) and suggests more extensive tissue damage. Histopathologically, rim+ lesions correspond to a subset of chronic active MS lesions with iron at the lesion edges retained by microglia/macrophages with a proinflammatory activation status (14, 15, 26), which has been linked to ongoing peri-plaque demyelination, gradual expansion of lesions, and the absence of remyelination (25, 27–29). Rim– lesions, in contrast, have been reported to show varying degrees of remyelination and a tendency to shrink (25, 27).

Interestingly, the current study found different distributional preferences between rim+ and rim– lesions at the individual level: more rim– lesions were located in juxtacortical and deep white matter areas, whereas a larger proportion of rim+ lesions were distributed in periventricular areas. Considering the distinct pathological characteristics of rim+ and rim– subgroups, these distributional features may facilitate the presentation of large and confluent periventricular lesions over time. Both the current study and a previous study confirmed larger lesion volumes in periventricular areas than in other regions (16). Our findings differ from those of a previous study, which showed similar portions of rim– lesions located near the ventricles and the cortex. In that study, comparisons were made at the lesion level and might not reflect the situations of individuals (30).

Longitudinal MRI studies demonstrate that the volume difference between rim+ and rim– lesions is significant even at their first appearance (27, 31), with a longer QSM persistence time in rim+ lesions as well as a slower growth rate and cubic decay of susceptibility. In addition, the QSM patterns of the two subgroups predominantly remained stationary for prolonged time periods. These findings may indicate distinct pathological courses of iron accumulation and removal during lesion formation and differing clinical consequences between rim+ and rim– lesions. The trigger for iron accumulation in activated macrophages in the rims of chronic active lesions is unknown, but based on our findings on lesion distribution, we speculate that certain spatially dependent factors may be involved.

Susceptibility contrast variations in demyelinated plaques are associated with MS pathology. In the early weeks of active demyelination, myelin debris is degraded by macrophages. The breakdown of myelin results in a rapid, slight increase in tissue susceptibility because of the loss of chemical bonds within diamagnetic myelin lipid layers (3, 32, 33). In the following months, MS plaques evolve into chronic lesions with a completely reconstituted BBB and no Gd enhancement. Lesion susceptibility continues to increase, but with a slower growth rate, which is probably attributable to iron deposition, and the susceptibility remains high and stable for years (13, 14, 25, 33). One speculation about the iron source is that during myelin digestion, paramagnetic iron may be released from ferritin (where it is primarily stored), taken up by macrophages, and deposited at the core or the periphery of the plaques (26, 34). Finally, in silent lesions, iron is completely removed and the elevated susceptibility correspondingly decays away (3, 13). These previous findings provide a foundation for interpreting the results of our study. First, QSM hyperintense lesions in RRMS patients, the focus of this study, may represent a subset of lesions that are mainly in the chronic stage and are likely to have iron deposition. Second, the susceptibility increase within lesions may result from the confounding effect of iron deposition and myelin loss.

MS, especially RRMS, features a wide spectrum of symptoms, severity, and response to therapy. Personalized therapy is important for disease modification, and the personalization of treatment relies on biomarkers correlated with clinical outcomes. Consistent evidence indicates that rim+ lesions are associated with poor clinical consequences (16, 25, 27, 35). This lesion subtype can be found in most MS patients and may therefore offer useful guidance for clinical practice to a limited extent. By subgrouping MS lesions based on QSM patterns and locations, we found a close relationship (β = 0.64) between the total burden of periventricular rim+ lesions and the ARR in our cohort. A large lesion volume reflects a relatively high content of iron release during demyelination. Iron clearance is a slow process and a high density of iron chronically concentrated at the lesion rim may cause repeated and long-lasting damage to the surrounding white matter and neurological deterioration in patients. This may explain the correlation found between the burden of rim+ lesions and the relapse rate. Our study also suggests that white matter damage is most symptomatic when located in periventricular areas.

Prior observations suggest that iron content, as measured by susceptibility, is higher in rim+ lesions than in rim– lesions (4, 30, 31), which is believed to be the cause of their larger volume or greater severity of tissue damage. In accordance with this, our study confirms that lesion susceptibility is positively related to lesion volume. Pathologically, iron rims present predominantly in slowly expanding lesions, much less in inactive lesions, and very little around fully remyelinated shadow plaques, indicating that iron may impair myelin repair (24, 25, 36). A thicker rim has been associated with a higher degree of demyelination (25, 37). These findings indicate that white matter damage severity could be iron level dependent, but the role of iron in MS lesion formation and disease progression is not clearly understood.

However, the current study did not show a significant association between clinical outcomes and average susceptibility of lesions after controlling for location. This result may be explained in part by the contribution of myelin loss, which may increase the susceptibility of MS lesions and compromise the linear relationship between iron and susceptibility. Another explanation is that the annual relapse rate (calculated retrospectively) and the susceptibility (measured cross-sectionally) were obtained in different stages of disease, although susceptibility in the chronic stage has been proven to be stable for several years. In contrast to an earlier longitudinal study showing that susceptibility values in rim+ lesions were higher at each time point than those in rim– lesions, our results demonstrated a lower average susceptibility in the rim+ subgroup of lesions, which may suggest lower iron content. However, in a previous study, only 32 lesions were included in their longitudinal observation, and lesion locations, which may have played a role in susceptibility, were not controlled in the analysis. On the other hand, susceptibility in our study was derived cross-sectionally from MS lesions at different ages. Previous data have indicated that the susceptibility of lesions declines over time and that the average variation range of susceptibility is as large as 20 ppb (31). Therefore, we hypothesize that a considerable portion of rim+ lesions were in old lesions, which would explain the low susceptibility in this subgroup of lesions. Longitudinal studies using techniques that can quantify the absolute contributions of iron and myelin to QSM signals are desired for a thorough understanding of iron-related damage in rim+ and rim– lesions, as well as the correlation of iron in these lesions with clinical outcomes.

In subtentorial areas, we found a positive correlation between ARR and the burden of rim– lesions, although their clinical consequences, based on supratentorial comparison with rim+ lesions, were modest. The infratentorial brain consists of abundant nuclei and compact fiber tracts ascending or descending to functionally connect the forebrain and spinal cord (38). In our study, the volume of lesions in subtentorial areas was relatively large compared with the lesion volumes in other regions, which may lead to the simultaneous involvement of multiple neural pathways and brainstem or cerebellum dysfunction (limb weakness, sensory loss, ataxia, etc.). Moreover, previous studies have demonstrated the direct extension of brainstem MS lesions toward the route entry/exit zone of cranial nerves, especially the trigeminal nerve (Supplementary Material), where these lesions have the potential to cause trigeminal neuralgia (39, 40). Overall, we suspect that subtentorial lesions may be more clinically relevant than lesions in supratentorial areas.

The results of this study suggest that MS lesion susceptibility is not uniform throughout the white matter. Juxtacortical lesions, whether in the rim+ or rim– subgroups, carry the highest susceptibility. This high susceptibility may result from large amounts of iron being liberated from myelin degradation products during demyelination. In support of this view, oligodendrocytes in the juxtacortical regions of normal or MS brains are known to be richer in iron than those in other regions (26). At the sites of demyelination, redox-inactive ferric (Fe^3+^) ions are released from the ferritin of oligodendrocytes and myelin sheaths and are exposed in the extracellular space, where they are converted to unbound ferrous (Fe^2+^) ions (15). The latter ions catalyze the production of reactive oxygen species. Ferrous iron is taken up by macrophages and subsequently transformed back into Fe^3+^ which causes detoxification of the tissue (26). Thus, our findings may indicate prominent oxidative tissue damage in juxtacortical plaques as a consequence of the high level of Fe^2+^ released. Although profound myelin loss may also contribute to high susceptibility in juxtacortical lesions, this assumption conflicts with the findings of a previous study, which showed a declining magnetization transfer ratio (MTR, reflecting myelin content) with increasing distance from the lateral ventricles and proximity to the cortex, suggesting a theoretically more pronounced susceptibility increase caused by periventricular myelin loss. In addition, we suggest that regional heterogeneity of iron accumulation in WM lesions should be considered in future studies. The clinical and pathological implications of high iron levels in juxtacortical lesions should be determined.

This study has limitations. First, the size of our patient cohort was small, and our findings are somewhat preliminary and warrant large-sample, longitudinal studies for confirmation. Second, our inference concerning the pathologic basis of QSM patterns was hypothesized based on prior evidence from studies correlating histopathology and imaging; the current study did not include its own histopathologic validation. Contrast-enhanced or longitudinal images were not collected to evaluate the possible pathologic status of the lesions. In addition, positive susceptibility values with respect to water are suggested to be a valid criterion to identify rims that contain real iron deposition (41). This process was not performed in this study because the ROIs drawn on QSM did not separate the core and rim areas of lesions, owing to the lack of sufficient image resolution. Moreover, only MS patients with a relapsing–remitting course were enrolled; this restriction may limit the generalizability of the results. Future studies comparing different clinical subtypes of MS would be desirable to clarify the contribution of lesion locations to clinical status. Finally, because of the small sample size, we did not divide participants into subgroups based on relapse status, which is closely related to EDSS scores in RRMS. This may be one reason why the correlation analysis found no variables that were significantly correlated with EDSS in our study. Another reason could be that spinal cord lesions, which correlate with physical disability more strongly than brain lesions, were not evaluated in the current study.

## Conclusion

Our study confirms the differences between the rim+ and rim– subgroups in terms of distributional patterns, susceptibility, extent of tissue damage, and clinical relevance. Lesion distribution holds important prognostic value in MS. A high number or lesion burden of periventricular rim+ lesions or subtentorial lesions is associated with frequent clinical relapses.

## Data Availability Statement

The original contributions presented in the study are included in the article/Supplementary Material, further inquiries can be directed to the corresponding author.

## Ethics Statement

The studies involving human participants were reviewed and approved by Ethics committee of the Third Affiliated Hospital of Sun Yat-sen university. The patients/participants provided their written informed consent to participate in this study.

## Author Contributions

SC and WQ: study concepts and design and manuscript revision. ZG and LL: data analysis, statistical analysis, and manuscript preparation. TL and YS: clinical data acquisition and analysis and manuscript review. LZ, KZ, and LF: imaging data acquisition and manuscript review. All authors contributed to the article and approved the submitted version.

## Conflict of Interest

The authors declare that the research was conducted in the absence of any commercial or financial relationships that could be construed as a potential conflict of interest.
