# Supplementary material for: The Distributional Characteristics of Multiple Sclerosis Lesions on Quantitative Susceptibility Mapping and Their Correlation With Clinical Severity
Source: Front Neurol. 2021 Jul 9;12:647519. doi: 10.3389/fneur.2021.647519 (PMC8299522; doi:10.3389/fneur.2021.647519)
Supplement: Supplementary file 1 [file Table_1.DOCX]

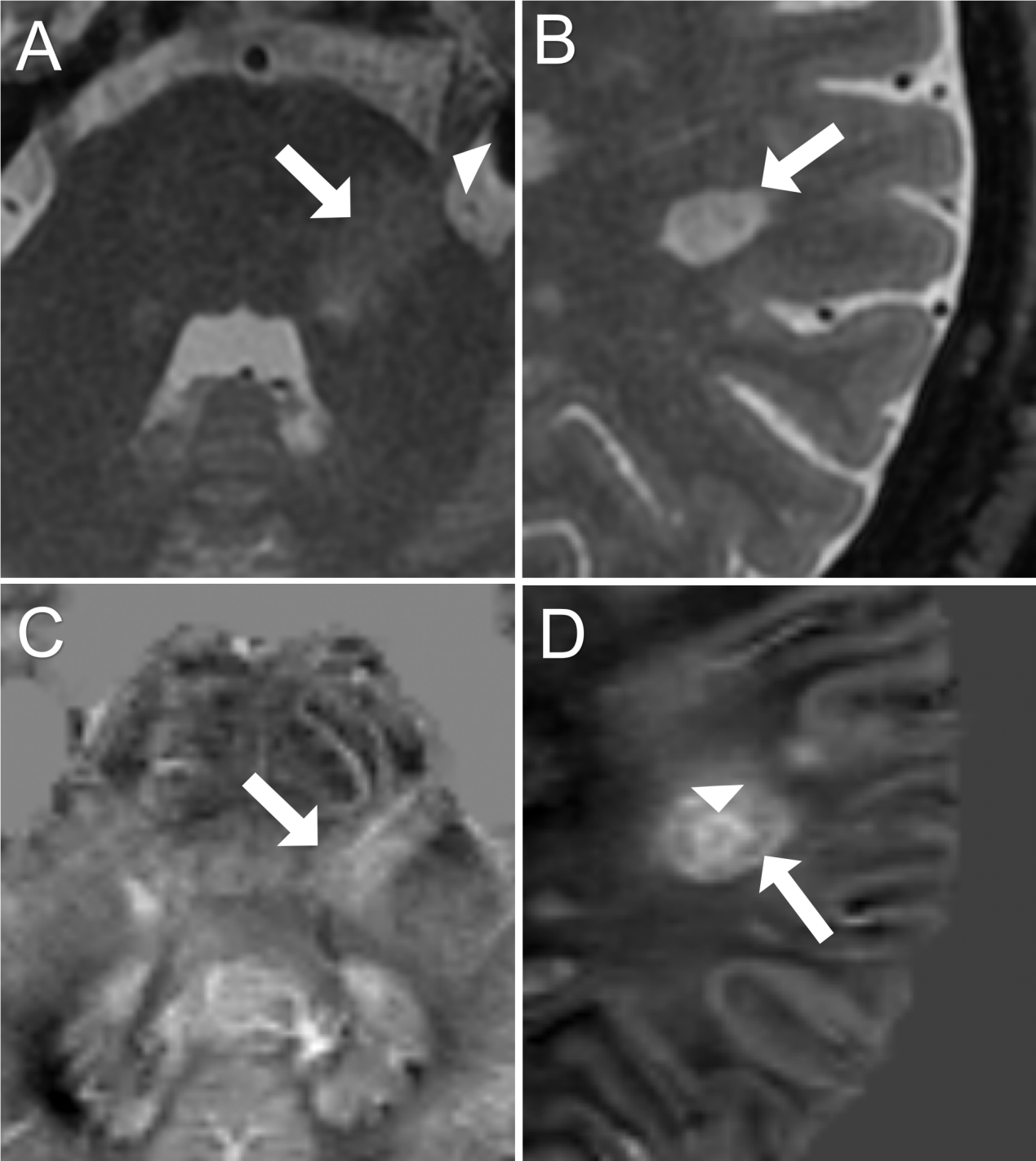


Figure 1. QSM appearances of MS lesions

A and C, a subtentorial MS lesions. The lesion is hyperintense on T2WI (**arrow, A**) and QSM (**arrow, C**), and the route exit zone of the trigeminal nerves is involved (**arrowhead, A**). T2WI demonstrate a juxtacortical MS lesion (**arrow, B**) with hyperintense rim on QSM (**arrow, D**). A ring-like QSM hyperintense was visualized at the centre of the rim+ lesion (**arrowhead, D**), demonstrating a “concentric ring” appearance.
